# Risk Factors, Prognosis, Influence on the Offspring, and Genetic Architecture of Perinatal Depression Classified Based on the Depressive Symptom Trajectory

**DOI:** 10.1155/2024/6622666

**Published:** 2024-03-15

**Authors:** Hisashi Ohseto, Ippei Takahashi, Akira Narita, Taku Obara, Mami Ishikuro, Natsuko Kobayashi, Saya Kikuchi, Xue Li, Aoi Noda, Keiko Murakami, Gen Tamiya, Junichi Sugawara, Hiroaki Tomita, Shinichi Kuriyama

**Affiliations:** ^1^Graduate School of Medicine, Tohoku University, Sendai 980-8575, Japan; ^2^Tohoku Medical Megabank Organization, Tohoku University, Sendai 980-8573, Japan; ^3^Tohoku University Hospital, Tohoku University, Sendai 980-8574, Japan; ^4^RIKEN Center for Advanced Intelligence Project, Tokyo 103-0027, Japan; ^5^Suzuki Memorial Hospital, Iwanuma 989-2427, Japan; ^6^International Research Institute of Disaster Science, Tohoku University, Sendai 980-8572, Japan

## Abstract

This study is aimed at revealing the risk factors, prognosis, influence on offspring, and genetic architecture of perinatal depression (PD) classified based on the depressive symptom trajectory. Pregnant women with no history of major depressive disorder (MDD) were recruited and followed up with their offspring from 1 to 5 years postpartum. Using four self-report questionnaires in the perinatal period, PD was classified into four subtypes: pregnancy, early postpartum, late postpartum, and chronic PD. Risk factors, depressive symptom trajectory from 1 to 5 years postpartum, and child behavior problems were compared among the four PD subtypes. Genome-wide association studies (GWASs) were conducted for each subtype. The relationships between the PD subtypes and polygenic risk scores (PRS) for MDD, a psychiatric disorder, and premenstrual syndrome (PMS), a hormonal disorder, were examined. Among 12,338 participants, 1,145 (9.3%) developed pregnancy PD, 856 (6.9%) developed early postpartum PD, 382 (3.1%) developed late postpartum PD, and 1,048 (8.5%) developed chronic PD. Depressive symptoms decreased to 61.0%–73.3% in the 5 years postpartum. The relationship between risk factors and PD varied based on the PD subtype. Additionally, chronic PD increased the risk of child behavior problems by 2- to 3-fold. The GWASs uncovered five significant variants in different loci depending on PD subtypes, suggesting a subtype-specific genetic architecture. The PRS for MDD was related to pregnancy, early postpartum, and chronic PD, while that for PMS was related to late postpartum PD. It was concluded that PD is heterogeneous depending on the depressive symptom trajectory. Thus, specific prevention and treatment strategies are needed.

## 1. Introduction

Perinatal depression (PD) is characterized by depressive symptoms emerging during pregnancy or postpartum [1], affecting approximately 15%–20% of women [2, 3], resulting in poor family relationships [4], poor infant care [5], and suicidal ideation [6]. Regarding the influence of PD on the offspring, large studies have noted child behavioral problems [7, 8]; however, this observation is inconsistent [9]. The pathophysiological mechanism of PD is expected to be similar to that of major depressive disorders (MDD). However, environmental and physiological changes specific to the perinatal period make it more complex [10]. Clinically, PD is often treated similarly to MDD; however, given its distinct biological background and impact on mothers and children, treatment based on PD-specific etiology is warranted [11].

Focusing on the depressive symptom trajectory is one approach to elucidate the mechanisms of PD. Studies that focused on environmental risk factors for PD classified based on the depressive symptom trajectory revealed its environmental heterogeneity [12, 13]. Given the unique stressors and physiologic conditions at each perinatal stage, assuming that the etiology and biological pathways also differ at each stage is reasonable. PD subtypes may be evident as the depressive symptom trajectory because they are expected to relate closely to perinatal hormone concentration trends or unique environmental factors in each stage of the perinatal period.

Genetically, the heritability of PD was 54% and 44% in twins and siblings [14], which were greater than those of MDD [15]. The largest genome-wide association study (GWAS) of PD [16], with 6,333 cases, observed only one significant genome-wide loci mapped on GULP1, which had not been previously reported to be associated with MDD. Additionally, the second largest GWAS [17], with 3,804 cases, observed no significant loci. In 2023, GWAS meta-analysis [18] was performed in 20 cohorts worldwide with 18,770 cases, but no significant loci were observed. A rational interpretation is that PD has various subtypes; i.e., it is heterogeneous. Thus, it is conceivable that genetic effects have been diluted in previous GWASs and have not been significant despite PD having higher heritability than MDD. Classifying PD into more homogeneous subtypes may increase the detection power in genomic studies.

In a genomic study [17], PD subtypes classified based on the depressive symptom trajectory were variably associated with polygenic risk score (PRS) for other psychiatric disorders. Additionally, previous studies [19] have highlighted that premenstrual syndrome (PMS), primarily caused by sex hormone disturbances, and MDD share a common etiology, suggesting that perinatal sex hormone disturbances may be involved in PD development. Therefore, we hypothesized that certain PD subtypes are genetically associated with the psychopathic predisposition as measured by PRS for MDD, while other PD subtypes would be associated with susceptibility to sex hormone disturbance as measured by PRS for PMS.

Here, we conducted a comprehensive study to elucidate PD's pathogenesis and confirmed that PD subtypes with different depressive symptom trajectory have different genetic and environmental etiologies. Four subtypes of PD, pregnancy, early postpartum, late postpartum, and chronic PD, were diagnosed prospectively. Five-year depressive symptom trends and child behavior problems at age five were compared. Additionally, with a focus on the genetic architecture of PD, we conducted GWAS and PRS analysis for each subtype.

## 2. Materials and Methods

### 2.1. Participants

The Tohoku Medical Megabank Project Birth and Three-Generation (TMM BirThree) Cohort Study [20] was initiated in July 2013 after the Great East Japan Earthquake to collect information on perinatal exposure, subsequent disorder development, and genomics to achieve personalized medicine and health management. From over 50 obstetric clinics and hospitals in the Miyagi Prefecture, Japan, 23,406 pregnant women were recruited. We excluded 232 participants who withdrew consent, 369 with MDD history or currently taking selective serotonin reuptake inhibitors, 192 with abortion or stillbirth, and 9,918 who did not complete all four perinatal depressive symptom questionnaires. Regarding the 357 participants who participated in the TMM BirThree Cohort Study more than once, only the first participation was included. Finally, 12,338 pregnant women were included in this study. All study participants provided informed consent, and ethics approval was obtained from the Ethics Committee of the Tohoku Medical Megabank Organization (2022-4-110).

### 2.2. Measures

Participants self-reported educational attainment (junior high school/high school, vocational school/junior college, or university/graduate), household income (≤4 million yen, 4–6 million yen, or ≥6 million yen), family history of MDD, acceptance of pregnancy at registration (good: “very happy”/“unexpected but happy,” or poor), social isolation (the Lubben Social Network Scale (LSNS-6) [21]<12 or not), marital status (unmarried or married), and neuroticism trait via the short-form Eysenck Personality Questionnaire Revised [22]. Overweight (prepregnancy body mass index > 25 kg/m^2^ or not), neonate sex, parity (primipara or not), multiple births, delivery mode, and pregnancy complications (fetal growth restriction, threatened preterm delivery, and hypertensive disorders of pregnancy) were obtained from the medical records. Participants responded to the child behavior checklist (CBCL) [23] when their children were 5 years old, from which three composite scales (internalizing, externalizing, and total problems) were calculated. Participants answered the Kessler Psychological Distress Scale (K6) [24] twice during pregnancy, the Edinburgh Postnatal Depression Scale (EPDS) [25] at 1 and 6 months postpartum, and K6 annually thereafter. K6 measures psychological distress using six questionnaires ranging from 0 to 24 points. EPDS measures postnatal depressive symptoms consisting of 10 questionnaires of 0–30 points [24, 25]. In this study, individuals with K6 of ≥9 and an EPDS ≥ 9 were considered depressed based on previous studies [26, 27]. Due to the nature of our cohort, some participants had suffered psychological and physical damage from the earthquake, possibly confounding the relationship between risk factors and PD or PD and child development. Therefore, house damages (total or partial, or none) and acquaintance deaths due to the earthquake were also collected through a self-reported questionnaire.

### 2.3. PD Subtypes

**Figure 1:**
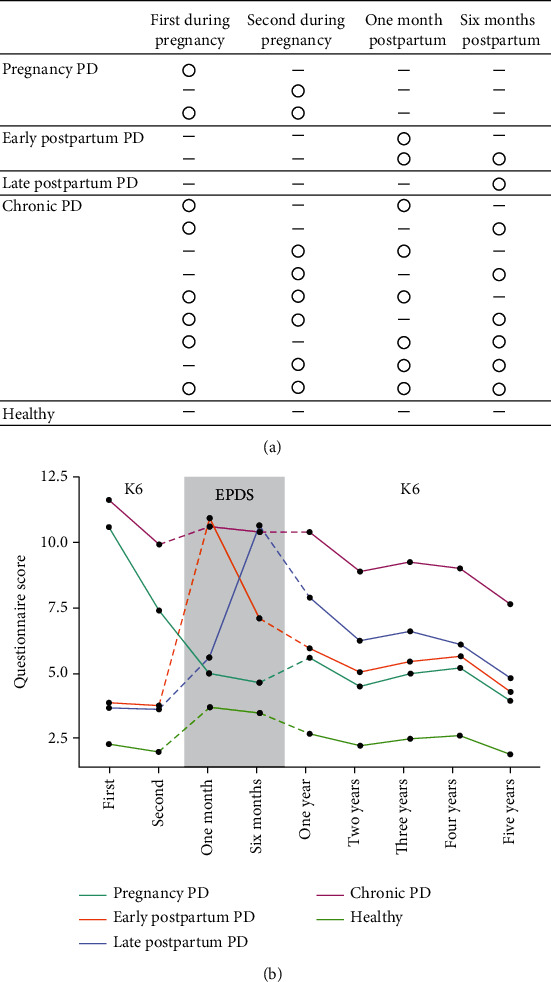
Definitions of the four PD subtypes and the depressive symptom trajectory from the perinatal period to 5 years postpartum. (a) According to four measurements of depressive symptoms during the perinatal period, four PD subtypes, pregnancy, early postpartum, late postpartum, and chronic PD, were defined. The “○” symbol represents “depressive,” and the “-” symbol represents “not depressive.” (b) Participants responded to K6 twice during pregnancy, EPDS at 1 month and 6 months postpartum, and K6 annually thereafter. Each line represents PD subtypes and healthy participants. The *x*-axis represents the time of the questionnaire, and the *y*-axis represents the mean questionnaire score in each group.

According to the depressive symptom trajectory measured by the four depressive symptom questionnaires, four PD subtypes were defined: pregnancy, early postpartum, late postpartum, and chronic PD (Figure 1(a)). Subtype-specific trajectories in the perinatal period were identified (Figure 1(b)). This definition was concordant with a previous study [12] that successfully demonstrated the heterogeneity of the environmental etiology of PD. Pregnancy PD was diagnosed when a pregnant woman was considered depressed at either or at both measurements during pregnancy but not at the postpartum measurements. Early postpartum PD was diagnosed when a pregnant woman without depression during pregnancy was considered depressed at the 1-month postpartum measurement, regardless of whether she still showed symptoms at the 6-month postpartum measurement or not. Late postpartum PD was diagnosed when a pregnant woman without depression during pregnancy and at the 1-month postpartum measurement had become depressed at the 6-month postpartum measurement. Chronic PD was diagnosed when a pregnant woman became depressed at both or either of the two measurements during pregnancy and at both or either the two postpartum measurements. Pregnant women who were not depressed at any of the four scheduled measurements were considered healthy. Using these definitions, the trajectories of all participants were categorized into four PD subtypes, or as healthy (Figure 1(a)).

### 2.4. Genotyping and Quality Control

This is described in detail in the Supplementary Note. Briefly, participants were genotyped on two platforms: Affymetrix Axiom Japonica Array v2 (JPA v2) [28] and Japonica Array NEO (JPA NEO) [29]. Participants genotyped on the first platform were cohort A, and those genotyped on the second were cohort B. In the genome-wide association study (GWAS), cohort A included 6,562 individuals with 11,737,866 variants, whereas cohort B included 5,098 individuals with 11,747,153 variants after imputation and standard quality control.

### 2.5. Statistical Analysis

Basic characteristics were compared among PD subtypes using the chi-square test for categorical variables and analysis of variance for continuous variables. The mean K6 scores at 1, 2, 3, 4, and 5 years postpartum were plotted for each PD subtype along with perinatal depressive symptom scores (K6 and EPDS) to reveal the long-term trajectory of depressive symptoms.

In risk factor analysis, risk factors for PD (age, educational attainment, household income, family history of MDD, acceptance of pregnancy, social isolation, marital status, neuroticism trait, house damages due to the earthquake, acquaintance deaths due to the earthquake, overweight, neonate sex, primipara, multiple births, delivery mode, and pregnancy complications) were compared using multinomial logistic regression analysis with healthy controls and each PD subtype as a case. In analyzing child behavior, CBCL scores were dichotomized based on whether the standardized *T*-score [23] was ≥64 for the three composite scales. Multiple logistic regression was applied with adjustment for those in risk factor analysis after excluding multiple births. Multiple imputation [30] was applied to missing values in risk factors, and the results were subsequently integrated.

GWASs were performed for each PD subtype and for “any PD” defined as any PD subtypes using GCTA (1.94.0) [31] with a generalized linear mixed model adjusted for age and ten principal components in cohorts A and B. Twenty-eight outlier individuals detected by first and second principal components were excluded. The results from both cohorts were meta-analyzed using METAL [32]. Genome-wide statistical significance was set at the conventional *P* value threshold of <5 × 10^−8^. Clumping was applied using PRSice-2 (2.3.5) [33] to determine independent variants. GWAS results were reported for all subtypes for significant or top-hit variants. For each reported variant, perinatal depressive symptom trajectories (K6 scores during pregnancy and EPDS scores at the 1- and 6-month postpartum measurements) were plotted according to genotypes to identify longitudinal effects. Fine mapping was performed using FOCUS for significant or top-hit variants [34]. FOCUS is a fine-mapping method that estimates posterior inclusion probability (PIP) for each gene to explain observed association signals from the transcriptome-wide association study utilizing GWAS summary data, expression weights, and the linkage disequilibrium among all SNPs in a region of association. Precomputed expression quantitative trait locus weights were used according to the original study [34]. The heritability of all PD subtypes and genetic correlations of all pairs of PD subtypes were estimated using LDSC [35].

PRS were calculated separately in cohorts A and B for MDD and PMS (Supplementary Note). The prevalence of each PD subtype was compared with the PRS quintiles using multiple logistic regression, with quintile 1 (lowest genetic load) as a reference, which was adjusted for age and five principal components. After that, the results for cohorts A and B were merged using inverse-variance weighting, and only merged results were reported. P for trends were compared among PRS with different *P* value thresholds (0.5, 0.1, 0.05, 0.01, and 0.005) for inclusion of variants to confirm the relationship's robustness. Additionally, to determine the effect of the genetic load of each phenotype on the trajectory of perinatal depressive symptoms, K6 scores during pregnancy and EPDS scores at the 1- and 6-month postpartum measurements were plotted for each PRS quintile after merging both cohorts, considering that the same quintiles had the same genetic load.

A series of sensitivity analyses were performed. First is to investigate the validity of the PD definition. Risk factor and PRS analyses were re-examined based on two additional PD definitions. According to previous studies, one defines “depressed” as K6 of ≥5, and the other defines it as K6 of ≥13 [24, 36, 37]. Second, those who became pregnant at the time they responded to the K6 at the 6 months postpartum were excluded, and risk factor and PRS analyses were performed again. Third, to account for the effect of current maternal depression on CBCL responses, the K6 score at the five-year postpartum measurement was added to the covariates in the child behavior analysis. Fourth, PRS for MDD was calculated from summary statistics of MDD GWAS meta-analysis, including the Japanese population [38], as opposed to the main analysis that included only the European population (Supplementary Note), and PRS analysis was performed again.

All analyses were conducted in R (4.1.0) unless otherwise noted. We considered two-tailed *P* values of 0.05 as significant.

## 3. Results

The timing of the four questionnaire responses (median [interquartile range]) was at 17.7 [14.4–21.0] and 27.3 [24.4–30.3] weeks of gestation and 4.4 [4.0–4.9] and 27.7 [26.0–34.1] weeks postpartum. Out of 12,338 participants, 1,145 (9.3%) developed pregnancy PD, 856 (6.9%) developed early postpartum PD, 382 (3.1%) developed late postpartum PD, and 1,048 (8.5%) developed chronic PD. Most covariates, except for acquaintance deaths and house damages during the earthquake, multiple births, delivery mode, and threatened preterm delivery differed among healthy participants and those with PD (Table 1). Regarding prognosis, at five years postpartum, the follow-up rates between PD and healthy participants were similar. Depressive symptom scores decreased to 61.0%–73.3% of that in the first year. However, participants with PD still exhibited high depressive symptoms (Figure 1(b) and Supplementary Table 1). Chronic PD scored approximately 3–5 points higher than other PD subtypes (Supplementary Table 1).

Risk factor analysis revealed that each PD subtype has a different relationship with risk factors and is largely consistent across sensitivity analyses (Supplementary Table 2). Older age was negatively associated with pregnancy and chronic PD, and lower household income was positively associated with early postpartum PD. However, no relationship was observed between education level and PD. Moreover, family history of MDD was positively associated with early postpartum and chronic PD, while poor acceptance was positively associated with PD, especially chronic PD. Social isolation and neuroticism traits were positively related to all PD subtypes. The relationship between unmarried status and PD changed across sensitivity analyses, with a relatively large confidence interval (CI). Acquaintance death was related to late postpartum PD. House damages from the earthquake, maternal overweight, multiple births, and cesarean section were unassociated with the PD subtypes. Male neonate was negatively associated with early postpartum PD. Primipara was a risk factor, except for late postpartum PD. Among the obstetric conditions, threatened prenatal delivery and hypertensive disorders of pregnancy were positively related to pregnancy and early postpartum PD and pregnancy PD, respectively, with moderate variation across sensitivity analyses.

After excluding multiple births and missing response cases, 6,618 mother-child pairs were eligible for child behavior analysis. All PD subtypes were positively related to all CBCL domain scores (Table 2). For instance, chronic PD was associated with a 2- to 3-fold risk of child behavior problems (odds ratio (OR) and 95% CI: 2.59 (2.01 to 3.33), 2.94 (2.27 to 3.80), and 3.26 (2.53 to 4.20) for internalizing, externalizing, and total problems, respectively), which remained significant after adjusting for the maternal depressive state when responding to CBCL. Early and late postpartum PD more strongly affected internalizing than external problem. However, this was not the case in pregnancy and chronic PD (Table 2).

**Figure 2:**
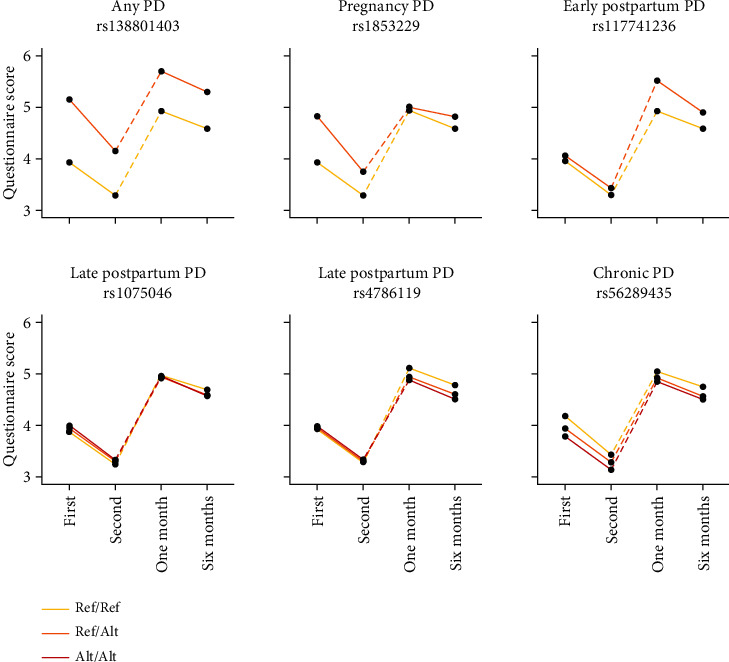
Perinatal depressive symptom trajectories according to genotypes of reported variants. Ref: reference allele; Alt: alternative allele. K6 scores obtained twice during pregnancy and EPDS scores obtained at 1 and 6 months postpartum were plotted according to genotypes of six reported variants. For variants with alternative allele frequencies smaller than 0.1, homozygous participants for the alternative alleles were included with heterozygous participants.

In the GWAS meta-analysis (Table 3), five variants located on an intron of CBR3-AS1 (rs138801403), an intron of PTPRD (rs1853229), an intron of CDH12 (rs1075046), an intron of RBFOX1 (rs4786119), and an intergenic between LOC100129620 and PLPPR4 (rs56289435) were significant for the relationship with any PD subtype (*P* = 3.4 × 10^−8^), pregnancy PD (*P* = 2.1 × 10^−8^), late postpartum PD (*P* = 3.7 × 10^−9^), late postpartum PD (*P* = 5.8 × 10^−9^), and chronic PD (*P* = 3.9 × 10^−8^), respectively. The top-hit variant of early postpartum PD (*P* = 6.5 × 10^−8^) was rs117741236, located on an intron of TGM5. Each variant exhibited a distinct longitudinal pattern specific to each genotype (Figure 2 and Supplementary Table 3). For instance, in the case of rs1853229, which was significant in pregnancy PD, participants who were heterozygous had higher depression scores during pregnancy than those who were homozygous for the major alleles. In fine mapping using FOCUS, the highest PIP for each variant was lnc-CBR3-1:11 in the aorta on chromosome 21 in any PD (PIP = 6.9%), LOC101928797 in fibroblasts on chromosome 9 in pregnancy PD (PIP = 10.8%), TUBGCP4 in basal ganglia (PIP = 5.3%) on chromosome 15 in early postpartum PD, CDH12 in dorsolateral prefrontal cortex on chromosome 5 in late postpartum PD, RBFOX1 in tibial nerve (PIP = 10.1%) on chromosome 16 in postpartum PD, and PLPPR4 in the testis (PIP = 11.1%) on chromosome 1 in chronic PD. Heritability estimates were 0.062 (standard error = 0.037), 0.037 (0.043), 0.010 (0.046), 0.078 (0.046), and 0.043 (0.046) for any PD subtype, pregnancy PD, early postpartum PD, late postpartum PD, and chronic PD, respectively. The genetic correlation was statistically significant only for any PD and chronic PD pairs (Supplementary Table 4; *r*_g_ = 0.73, SE = 0.36, *P* = 0.04).

**Figure 3:**
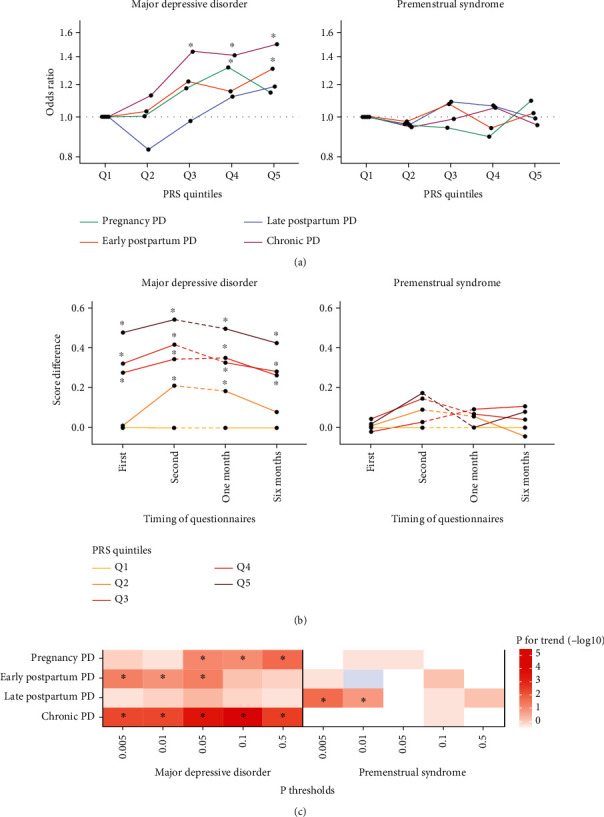
PRS analysis. (a) The prevalence of each PD subtype was compared among PRS (left: MDD; right: PMS) quintiles using multiple logistic regression, considering quintile 1 (lowest genetic load) as a reference, adjusted for age and five principal components. The *x*-axis is the PRS quintiles, and the *y*-axis is the odds ratio. The asterisk indicates statistical significance (*P* < 0.05). (b) K6 scores obtained twice during pregnancy and EPDS scores obtained at 1 and 6 months postpartum were plotted by PRS (left: MDD; right: PMS) quintiles. The *x*-axis represents the time of the questionnaires, and the *y*-axis represents the mean difference between questionnaire scores in each quintile group and the healthy control. (c) The heat map indicates the P for trend calculated using PRS with different *P* value thresholds (0.5, 0.1, 0.05, 0.01, and 0.005). The *x*-axis indicates PRS phenotype and *P* value thresholds, and the *y*-axis represents PD subtypes.

PRS analysis revealed a significant positive relationship between genetic susceptibility to MDD and pregnancy, early postpartum, and chronic PD (Figure 3(a) and Supplementary Table 3), largely consistent with the different *P* value thresholds (Figure 3(c)). Those with high PRS for MDD had high depressive symptoms throughout the perinatal period. In contrast, those with high PRS for PMS displayed no apparent symptom trend (Figure 3(b)). PRS for PMS with *P* values 0.005 and 0.01 were related to late postpartum PD (Figure 3(c)). Each PD subtype was not associated with PRS for MDD from a meta-analysis including Japanese population (Supplementary Table 5). The results were largely consistent across sensitivity analyses (Supplementary Table 5).

## 4. Discussion

PD classified based on the depressive symptom trajectory had different relationships with risk factors such as socioeconomic status, maternal psychological status, and obstetric status. Each PD subtype, especially chronic PD, was a significant risk factor for child behavior problems at five years postpartum. The GWASs for each subtype identified four susceptibility loci that were not identified before subtyping. PRS analysis revealed the relationship between PRS for MDD and pregnancy, early postpartum, and chronic PD and between PRS for PMS and late postpartum PD.

The score trajectory displayed a gradual decrease in depressive scores for participants with PD. However, it remained at approximately 70% of the first year, even five years postpartum. Previous studies [39, 40] examining depressive symptoms for 1–3 years in dozens to hundreds of patients with PD also observed that depressive symptoms attenuated but remained at follow-up. This study confirms this result in a larger population over a prolonged follow-up, demonstrating the need for long-term care for patients with PD.

Risk factor analysis revealed a clear difference in PD subtypes, mostly consistent with other PD definitions in a sensitivity analysis. A previous study [12] that also focused on risk factors for PD classified based on the depressive symptom trajectory revealed largely consistent results with our result concerning them being risk or protective factors. However, the characteristics of PD subtypes differed. Older age was more associated with late postpartum PD than early postpartum PD in this study. Nonetheless, the relationship was reversed in the previous study [12]. After delivery, the mother's social circumstance changes gradually, but her physiologic status rapidly normalizes. PD detected early may have more specific physiologic features; therefore, EPDS measurement timing is a critical factor in defining the pathophysiology, and its difference (at 6 weeks and 6 months postpartum in the previous study [12], whereas at 4 weeks and 6 months postpartum in this study) could explain the different results between the previous [12] and this study. It is also important to note the difference in population (Swedes vs. Japanese), measurement (EPDS only vs. K6 and EPDS), and statistical method (univariate vs. multivariate).

Child behavior analysis revealed the prominent risk of PD for child behavior problems, which was attenuated but remained significant after adjusting for maternal depressive symptoms when responding to CBCL. In a previous Japanese study of 1,199 children [41], children whose mothers had depressive symptoms both during pregnancy and postpartum had a fivefold increased risk of childhood emotional symptoms with CBCL, compared to a twofold increase with either pregnancy or postpartum alone. Moreover, a small previous study [42] observed that PD was related to child behavior problems, and this relationship was not necessarily dependent on the presence of current depressive symptoms in the mother, consistent with the findings of the current study.

The GWASs uncovered five significant variants. CBR3-AS1, which was related to any PD, was not significant in the previous GWASs for any psychiatric disorders to date. However, PTPRD was reported in the largest East Asian GWAS for MDD [43], and RBFOX1 was reported in the largest European GWAS for MDD [44]. CDH12 was reported in the GWAS for suicide [45], and PLPPR4 was reported in the GWAS for antidepressant response [46]. Despite the small number of cases compared to previous GWAS [17], the GWASs for each PD subtype could identify biologically relevant loci, suggesting that a GWAS with small but homogeneous subgroups can detect susceptibility loci [47]. Each variant was significant specifically for each subtype and had a characteristic depressive symptom trajectory based on genotype, reflecting each variant's distinctive pathological mechanism. Fine mapping suggested the involvement of several genes; however, PIP was small and did not show strong evidence of causality. The small sample size of the current study makes distinguishing subtype-specific findings from random detection challenging. Therefore, we hope that larger studies with biological reproducibility will confirm our findings and uncover new loci in the future. Heritability was estimated at 0.01-0.078, which was relatively small compared with that in previous GWAS [18], estimated at 0.17 in the East Asian population. In addition to the small sample size, our assessment of PD was based solely on self-report and may be susceptible to environmental factors. Consequently, the estimates of genetic correlations were also difficult to interpret with high variance. These results indicate that attempts to split outcomes to create a more homogeneous population are subject to the challenge of decreasing sample size, especially in post-GWAS.

PRS analysis revealed the genetic relationship between MDD and pregnancy, early postpartum, and chronic PD, a notable finding in our study. The previous study [48] revealed that PRS for MDD was associated with prenatal and postpartum depressive symptoms and that perinatal depressive symptoms mediated 57.9%–88.0% of the effect of PRS on postpartum depression symptoms. Taken together with our result, genetic susceptibility to MDD affects pregnancy and early postpartum depressive symptoms, and late postpartum depressive symptoms are more dependent on prior depressive symptoms. PRS for PMS was not associated with pregnancy, early postpartum, or chronic PD, which is inconsistent with our hypothesis that perinatal sex hormone disturbance causes psychiatric symptoms, especially in pregnancies genetically susceptible to sex hormone disturbance. Additionally, the relationship between PRS for PMS and late postpartum PD may be explained by PMS symptoms after the postpartum menstrual cycle resumes, indicating the unique pathology of late postpartum PD.

Our study has at least three clinical and research implications. First, PD caused persistent maternal depressive symptoms and child behavioral problems even 5 years postpartum, suggesting the need for long-term care such as life support, environmental adjustment, and the affected mothers learning to cope with stress. Particularly, the high-risk mothers indicated in this study, such as young, low-income, unmarried, and socially isolated, require intensive care. Second, PD subtypes classified based on the depressive symptom trajectory have unique prognoses, risk factors, influence on the offspring, and genetic architecture that will provide clues to new prevention and treatment strategies. MDD medication, such as selective serotonin reuptake inhibitors, may be less effective in late postpartum PD than in other PD subtypes. Third, the depressive symptoms score trajectory differed across the perinatal period; this can be used in clinical practice to identify high-risk periods of PD and provide focused care.

The strength of this study is that it is the first to conduct GWAS and PRS analysis for PD classified based on the prospectively assessed depressive symptom trajectory. Only one previous study [17] focused on PD subtypes classified based on the depressive symptom trajectory and performed a GWAS and PRS analysis. However, diagnosis and identification of depressive symptom trajectory were retrospective, and the genetic relationship with PMS was not investigated. The prolonged follow-up and assessment of influence on the offspring in the current study allow for comprehensiveness. Our cohort recruited about half of the newborns in Miyagi Prefecture in 2016, which led to a relatively small sampling bias. The percentage of EPDS above the cutoff was similar to that of the general population [20].

Despite these strengths and the sensitivity analysis series, this study has at least five limitations. First, our definition of PD has not been validated. K6 during pregnancy is not a common measure of depressive symptoms, and postpartum depressive symptoms have only been assessed twice (at 1 and 6 months postpartum). We confirmed the robustness of our result using a different K6 cutoff; however, our definition may miss the dynamics of depressive symptoms up to four weeks postpartum. Second, we used the GWAS results from a European rather than Japanese population to calculate the PRS for MDD in the main analysis. Thus, the PRS may not accurately reflect the polygenic loadings for MDD. The GWAS meta-analysis including the Japanese population has a smaller sample size (cases < 15,000, including only 836 Japanese vs. 246,363 cases in the main analysis). Therefore, its validity is questionable. Third, less than 1% of participants were pregnant at the time they responded to the K6 at 6 months postpartum, which may have resulted in misclassification given that it is impossible to distinguish postpartum depressive symptoms from current pregnancy depressive symptoms. However, sensitivity analyses excluding these participants yielded comparable results, indicating robustness. Fourth, PD diagnosis and assessment of children depend on mothers' self-reporting. Finally, some significant or top-hit variants had minor allele frequencies as low as ≤5%, making false positives more likely due to the available sample size.

## 5. Conclusions

PD subtypes classified based on the depressive symptom trajectory revealed different risk factors, prognosis, and influence on the offspring. The GWASs for each subtype identified four susceptibility loci that were not identified before subtyping. PRS for MDD was related to pregnancy, early postpartum, and chronic PD, while PRS for PMS was related to late postpartum PD, suggesting subtype-specific genetic architecture.

## Figures and Tables

**Table 1:** Baseline characteristics and child behavior problems at five years postpartum.

	Healthy (*n* = 8,907)		Pregnancy PD (*n* = 1,145)		Early postpartum PD (*n* = 856)		Late postpartum PD (*n* = 382)		Chronic PD (*n* = 1,048)		*P* value
	*n*	%	*n*	%	*n*	%	*n*	%	*n*	%	
Age at delivery											<0.001
≤30 years old	2,485	27.9	436	38.1	267	31.2	133	34.8	439	41.9	
30–35 years old	3,402	38.2	411	35.9	347	40.5	139	36.4	369	35.2	
≥35 years old	3,020	33.9	298	26.0	242	28.3	110	28.8	240	22.9	
Education attainment											<0.001
Junior high school/high school	2,298	31.6	308	33.6	222	32.5	112	36.2	359	42.9	
Vocational school/junior college	2,887	39.7	357	39.0	280	41.0	111	35.9	285	34.1	
University/graduate	2,096	28.8	251	27.4	181	26.5	86	27.8	192	23.0	
Household income											<0.001
≤4 million yen	2,731	32.1	441	40.2	296	35.9	138	37.8	476	48.4	
4–6 million yen	4,610	54.2	522	47.6	454	55.1	191	52.3	430	43.7	
≥6 million yen	1,163	13.7	134	12.2	74	9.0	36	9.9	78	7.9	
Family history of MDD	479	6.5	85	9.2	69	10.0	31	10.0	82	9.7	<0.001
Poor acceptance of pregnancy	638	7.2	172	15.0	79	9.2	58	15.2	203	19.4	<0.001
Social isolation (LSNS ≤ 11)	1,417	16.0	258	22.7	218	25.7	92	24.2	382	36.6	<0.001
Unmarried	110	1.5	30	3.3	14	2.0	9	2.9	31	3.7	<0.001
Acquaintance deaths in the disaster	1,457	20.6	199	22.4	138	20.8	80	26.7	183	22.6	0.065
House damages in the disaster	762	10.4	118	12.9	74	10.8	37	12.1	83	10.0	0.190
Overweight	1,062	12.1	132	11.7	120	14.2	58	15.5	151	14.6	0.022
Male neonate	4,622	51.9	590	51.5	407	47.5	178	46.6	530	50.6	0.046
Primipara	3,284	36.9	511	44.6	470	54.9	137	35.9	471	44.9	<0.001
Multiple births	90	1.0	16	1.4	9	1.1	7	1.8	10	1.0	0.449
Cesarean section	2,103	23.6	283	24.7	212	24.8	86	22.5	235	22.4	0.651
Threatened preterm delivery	137	1.5	23	2.0	20	2.3	10	2.6	15	1.4	0.161
Fetal growth restriction	1,333	15.0	226	19.7	166	19.4	67	17.5	182	17.4	<0.001
Hypertensive disorders of pregnancy	342	3.8	71	6.2	50	5.9	20	5.2	47	4.5	<0.001
Neuroticism	Mean	SD	Mean	SD	Mean	SD	Mean	SD	Mean	SD	<0.001
	4.99	2.8	7.85	2.7	7.28	2.6	7.25	2.8	9.34	2.1	
	*n*	%	*n*	%	*n*	%	*n*	%	*n*	%	
CBCL											
Internalizing score ≥ 7	369	7.7	96	15.7	75	16.7	33	17.1	138	25.2	<0.001
Externalizing score ≥ 12	356	7.4	92	15.0	57	12.7	26	13.5	142	25.9	<0.001
Total score ≥ 27	330	6.9	96	15.7	63	14.1	31	16.1	150	27.4	<0.001

PD: perinatal depression; SD: standard deviation; MDD: major depressive disorder; CBCL: child behavior checklist. *P* values were calculated using chi-square for categorical variables and analysis of variance for neuroticism score.

**Table 2:** Relationship between PD subtypes and child behavior problems at 5 years of age.

	Pregnancy PD			Early postpartum PD			Late postpartum PD			Chronic PD		
	OR	95% CI	*P* value	OR	95% CI	*P* value	OR	95% CI	*P* value	OR	95% CI	*P* value
Internalizing problems												
Crude	2.24	1.76, 2.86	<0.001	2.42	1.85, 3.17	<0.001	2.49	1.68, 3.67	<0.001	4.06	3.26, 5.06	<0.001
Risk factors adjusted	1.67	1.29, 2.16	<0.001	1.75	1.32, 2.32	<0.001	2.07	1.38, 3.09	<0.001	2.59	2.01, 3.33	<0.001
Risk factors and current K6 adjusted	1.50	1.15, 1.95	0.003	1.55	1.17, 2.07	0.002	1.70	1.13, 2.56	0.011	1.99	1.53, 2.60	<0.001
Externalizing problems												
Crude	2.22	1.73, 2.84	<0.001	1.83	1.36, 2.46	<0.001	1.95	1.27, 2.99	0.002	4.38	3.52, 5.46	<0.001
Risk factors adjusted	1.70	1.30, 2.23	<0.001	1.41	1.03, 1.93	0.030	1.70	1.10, 2.64	0.018	2.94	2.27, 3.80	<0.001
Risk factors and current K6 adjusted	1.56	1.19, 2.05	0.001	1.28	0.93, 1.75	0.126	1.44	0.92, 2.25	0.108	2.37	1.82, 3.10	<0.001
Total problems												
Crude	2.53	1.98, 3.23	<0.001	2.22	1.67, 2.97	<0.001	2.60	1.74, 3.88	<0.001	5.12	4.12, 6.38	<0.001
Risk factors adjusted	1.89	1.45, 2.45	<0.001	1.65	1.22, 2.24	0.001	2.18	1.44, 3.29	<0.001	3.26	2.53, 4.20	<0.001
Risk factors and current K6 adjusted	1.71	1.31, 2.24	<0.001	1.48	1.09, 2.01	0.012	1.82	1.20, 2.77	0.005	2.59	1.99, 3.37	<0.001

PD: perinatal depression; OR: odds ratio; CI: confidence interval; K6: Kessler Psychological Distress Scale. The cutoffs were 11, 16, and 39 for internalizing, externalizing, and total problems, respectively. Risk factors adjusted model included all variables in the risk factor analysis (age, educational attainment, household income, family history of major depressive disorder, acceptance of pregnancy, social isolation, marital status, neuroticism trait, house damages by the earthquake, acquaintance deaths during the earthquake, overweight, neonate sex, primipara, multiple births, delivery mode, and pregnancy complications) as covariates. Risk factors and current K6 adjusted model included K6 scores at 5 years postpartum in addition to the covariates in the risk factors adjusted model.

**Table 3:** Significant or top-hit variants in any PD or PD subtypes.

	Chr	RSID	Position	Ref	Alt	EA	Function	Gene	JPA v2				JPA NEO				Meta-analysis	
									EA freq.	Beta	SE	*P* value	EA freq.	Beta	SE	*P* value	*P* value	Direction
Any PD	21	rs138801403	37521946	G	A	A	ncRNA_intronic	CBR3–AS1	0.012	0.84	0.19	5.8 × 10^–6^	0.013	0.64	0.20	1.4 × 10^–3^	3.4 × 10^–8^	++
Pregnancy PD	9	rs1853229	10503904	C	T	T	Intronic	PTPRD	0.021	0.90	0.22	4.5 × 10^–5^	0.021	0.98	0.26	1.2 × 10^–4^	2.1 × 10^–8^	++
Early postpartum PD	15	rs117741236	43555394	A	G	G	Intronic	TGM5	0.023	1.01	0.24	3.2 × 10^−5^	0.027	0.90	0.26	5.5 × 10^−4^	6.5 × 10^–8^	++
Late postpartum PD	5	rs1075046	22852984	C	G	G	Intronic	CDH12	0.546	–0.31	0.10	2.2 × 10^–3^	0.549	–0.63	0.12	5.2 × 10^–8^	3.7 × 10^–9^	—
Late postpartum PD	16	rs4786119	6803436	T	C	C	Intronic	RBFOX1	0.615	–0.38	0.11	3.2 × 10^–4^	0.609	–0.57	0.12	2.3 × 10^–6^	5.8 × 10^–9^	—
Chronic PD	1	rs56289435	99692247	CACACAA	C	C	Intergenic	LOC100129620/PLPPR4	0.498	–0.20	0.07	3.1 × 10^–3^	0.500	–0.37	0.07	7.2 × 10^–7^	3.9 × 10^–8^	—

PD: perinatal depression; JPA v2: Japonica Array v2; JPA NEO: Japonica Array NEO; Ref: reference allele; Alt: alternative allele; EA: effect allele; EA freq: EA frequency; SE: standard error; ncRNA: noncoding RNA. The direction column indicates whether variants were positively or negatively associated with outcomes in each of the two cohorts.

## Data Availability

Summary GWAS statistics are publicly available at the Japanese Multi Omics Reference Panel website (https://jmorp.megabank.tohoku.ac.jp/). Individual genotyping results are available upon request after the approval of the Ethical Committee and the Materials and Information Distribution Review Committee of Tohoku Medical Megabank Organization.

## Additional Points

*Code Availability*. The code supporting this study's findings has been uploaded to GitHub. It is publicly accessible and can be found at https://github.com/HisashiOhseto/Genetic-architecture-of-perinatal-depression. Please contact the corresponding author for further inquiries or issues with accessing the code.

## Supplementary Materials

Supplementary Materials Supplementary Methods: Genotyping and quality control, polygenic risk score. Supplementary Tables: Supplementary Table 1: depressive symptom trajectory over a 5-year period. Supplementary Table 2: risk factor analysis using multinomial logistic regression. Supplementary Table 3: association of six GWAS significant or top-hit variants with PD subtypes. Supplementary Table 4: genetic correlations between PD subtypes. Supplementary Table 5: relationship between polygenic risk score for major depressive disorder and premenstrual syndrome and PD subtypes.
