# Supplementary material for: Risk Factors, Prognosis, Influence on the Offspring, and Genetic Architecture of Perinatal Depression Classified Based on the Depressive Symptom Trajectory
Source: Depress Anxiety. 2024 Mar 15;2024:6622666. doi: 10.1155/2024/6622666 (PMC11918876; doi:10.1155/2024/6622666)
Supplement: Supplementary Materials — Supplementary Methods: Genotyping and quality control, polygenic risk score. Supplementary Tables: Supplementary Table 1: depressive symptom trajectory over a 5-year period. Supplementary Table 2: risk factor analysis using multinomial logistic regression. Supplementary Table 3: association of six GWAS significant or top-hit variants with PD subtypes. Supplementary Table 4: genetic correlations between PD subtypes. Supplementary Table 5: relationship between polygenic risk score for major depressive disorder and premenstrual syndrome and PD subtypes. [file 6622666.f1.docx]

Supplementary Methods

Genotyping and quality control

Participants were genotyped using an Affymetrix Axiom Japonica Arrayv2 (JPA v2) or Japonica Array NEO (JPA NEO). Pre-imputation quality controls are detailed elsewhere[1].For imputation, pre-phasing was performed using SHAPEIT2. The phased genotypes were subsequently imputed using IMPUTE4[2]with the cross-imputed panel of 3.5KJPNv2[3] and 1KGP3[4] created using IMPUTE2[5] with-merge_ref_panels option. After imputation, variants with minor allele frequencies <0.01 and imputation information scores<0.4 were excluded. Finally, cohort A, genotyped using JPA v2, included 6,562 individuals with 11,737,866 variants, whilecohort B, genotyped using JPA NEO, included 5,098 individuals with 11,747,153 variants.

Polygenic risk score

Polygenic risk scores (PRS) for two phenotypes, including major depressive disorder (MDD) and premenstrual syndrome (PMS), werecalculated for each participant. The largest GWAS[6] summary statistics of MDDwere used for PRS calculation for MDD.GWAS summary statistics for PMSwere obtained by analyzingthe Tohoku Medical Megabank Project Community-Based Cohort Study (CommCohort)[7]. Participants in the CommCohort study answered the question: “Before menopause, did you have swelling, abdominal pain, back pain, loss of appetite, or emotional instability in the 3–10 days before menstruation?” and those who answered “yes” were considered to have had PMS. Using the same procedure as that used in the main analysis, 14,688 (7,100 cases) individuals with 11,737,866variants in JPA v2 were included in the GWAS,and summary statistics for PMS were obtained to calculate PRS. As a sensitivity analysis, GWAS summary statistics for MDD, includingthe Japanese population[8],were used to calculate an alternative PRS for MDD.

PRS was calculated using genome-wide clumping and thresholding methods described elsewhere [9].We restricted variants to HapMap3 following PRS-CS[10] and LDpred2[11] implementation methods. After standard quality control in the base and target data, clumping was conducted using plink (2.0)[12],with options --clump-p1: 1, --clump-r2: 0.1, and --clump-kb: 250. Subsequently, PRS was calculated with the P-value thresholds of 0.5, 0.1, 0.05, 0.01, and 0.005, respectively.A P-value threshold of 0.05 was used for the main PRS analysis.Other thresholds were used for calculating the P for trend to confirm the robustness of the main analysis (main text).

Supplementary Table 1. Depressive symptoms trajectory over a 5-year period

|  |  | Pregnancy | | | | |  | Postpartum | | | | | | | | | | | | | | | | | | | | | | | |
| --- | --- | --- | --- | --- | --- | --- | --- | --- | --- | --- | --- | --- | --- | --- | --- | --- | --- | --- | --- | --- | --- | --- | --- | --- | --- | --- | --- | --- | --- | --- | --- |
|  |  | K6 | | | | |  | EPDS | | | | |  | K6 | | | | | | | | | | | | | | | | | |
|  |  | First | |  | Second | |  | First month | |  | Sixth month | |  | First year | |  | Second year | | |  | Third year | | |  | Fourth year | | |  | Fifth year | | |
| n and follow-up rate (%) | | n | % |  | n | % |  | n | % |  | n | % |  | n | % |  | n | % |  |  | n | % |  |  | n | % |  |  | n | % |  |
|  | Pregnancy PD | 1,145 | 100.0 |  | 1,145 | 100.0 |  | 1,145 | 100.0 |  | 1,145 | 100.0 |  | 915 | 79.9 |  | 850 | 74.2 |  |  | 804 | 70.2 |  |  | 728 | 63.6 |  |  | 667 | 58.3 |  |
|  | Early postpartum PD | 856 | 100.0 |  | 856 | 100.0 |  | 856 | 100.0 |  | 856 | 100.0 |  | 679 | 79.3 |  | 617 | 72.1 |  |  | 598 | 69.9 |  |  | 537 | 62.7 |  |  | 481 | 56.2 |  |
|  | Late postpartum PD | 382 | 100.0 |  | 382 | 100.0 |  | 382 | 100.0 |  | 382 | 100.0 |  | 306 | 80.1 |  | 264 | 69.1 |  |  | 257 | 67.3 |  |  | 223 | 58.4 |  |  | 221 | 57.9 |  |
|  | Chronic PD | 1,048 | 100.0 |  | 1,048 | 100.0 |  | 1,048 | 100.0 |  | 1,048 | 100.0 |  | 831 | 79.3 |  | 757 | 72.2 |  |  | 695 | 66.3 |  |  | 651 | 62.1 |  |  | 598 | 57.1 |  |
|  | Healthy | 8,907 | 100.0 |  | 8,907 | 100.0 |  | 8,907 | 100.0 |  | 8,907 | 100.0 |  | 7,270 | 81.6 |  | 6,549 | 73.5 |  |  | 6,198 | 69.6 |  |  | 5,622 | 63.1 |  |  | 5,147 | 57.8 |  |
|  |  |  |  |  |  |  |  |  |  |  |  |  |  |  |  |  |  |  |  |  |  |  |  |  |  |  |  |  |  |  |  |
| Mean, SD, and rate to the first year (%) | | Mean | SD |  | Mean | SD |  | Mean | SD |  | Mean | SD |  | Mean | SD |  | Mean | SD | % |  | Mean | SD | % |  | Mean | SD | % |  | Mean | SD | % |
|  | Pregnancy PD | 10.6 | 4.5 |  | 7.4 | 4.3 |  | 5.0 | 2.2 |  | 4.6 | 2.1 |  | 5.6 | 4.4 |  | 4.5 | 4.2 | 80.2 |  | 5.0 | 4.5 | 89.1 |  | 5.2 | 4.6 | 93.1 |  | 3.9 | 3.7 | 70.3 |
|  | Early postpartum PD | 3.8 | 2.5 |  | 3.7 | 2.4 |  | 10.9 | 2.4 |  | 7.1 | 3.4 |  | 5.9 | 4.5 |  | 5.0 | 4.4 | 84.6 |  | 5.4 | 4.6 | 91.2 |  | 5.6 | 5.0 | 94.7 |  | 4.3 | 4.0 | 71.7 |
|  | Late postpartum PD | 3.7 | 2.5 |  | 3.6 | 2.5 |  | 5.6 | 2.0 |  | 10.6 | 2.5 |  | 7.8 | 4.9 |  | 6.2 | 4.7 | 79.2 |  | 6.6 | 4.9 | 83.7 |  | 6.1 | 4.8 | 77.5 |  | 4.8 | 3.8 | 61.0 |
|  | Chronic PD | 11.6 | 4.4 |  | 9.9 | 4.5 |  | 10.6 | 4.0 |  | 10.4 | 4.3 |  | 10.4 | 5.4 |  | 8.9 | 5.5 | 85.3 |  | 9.2 | 5.8 | 89.0 |  | 9.0 | 6.0 | 86.5 |  | 7.6 | 5.2 | 73.3 |
|  | Healthy | 2.2 | 2.3 |  | 2.0 | 2.1 |  | 3.7 | 2.1 |  | 3.4 | 2.0 |  | 2.7 | 2.9 |  | 2.2 | 2.9 | 82.6 |  | 2.5 | 3.1 | 92.6 |  | 2.6 | 3.3 | 96.8 |  | 1.9 | 2.6 | 70.7 |

K6: Kessler Psychological Distress Scale, EPDS: Edinburgh Postnatal Depression Scale, PD: Perinatal depression, SD: Standard deviation.

Supplementary Table 2. Risk factor analysis using multinomial logistic regression

|  |  | Pregnancy PD | | | | | Early postpartum PD | | | | | | Late postpartum PD | | | | | | Chronic PD | | | | | | |  |
| --- | --- | --- | --- | --- | --- | --- | --- | --- | --- | --- | --- | --- | --- | --- | --- | --- | --- | --- | --- | --- | --- | --- | --- | --- | --- | --- |
|  |  | OR | 95% CI | | | P-value | OR | | 95% CI | | | P-value | OR | | 95% CI | | | P-value | OR | | 95% CI | | | P-value | |  |
|  |  |  |  | | |  |  | |  | | |  |  | |  | | |  |  | |  | | |  | |  |
| Main definition | |  | |  |  | | |  | |  |  | | |  | |  |  | | |  | |  |  | | | |
|  | Age at delivery, years (30–35 vs. < 30) | 0.82 | 0.70, 0.96 | | | 0.012 | 1.18 | | 0.98, 1.41 | | | 0.076 | 0.84 | | 0.65, 1.08 | | | 0.170 | 0.84 | | 0.70, 1.00 | | | 0.051 | |  |
|  | Age at delivery, years (> 35 vs. < 30) | 0.72 | 0.60, 0.86 | | | < 0.001 | 1.03 | | 0.85, 1.27 | | | 0.743 | 0.79 | | 0.60, 1.05 | | | 0.102 | 0.71 | | 0.58, 0.88 | | | 0.002 | |  |
|  | Education level (middle vs. high) | 0.96 | 0.80, 1.15 | | | 0.657 | 1.07 | | 0.88, 1.31 | | | 0.499 | 0.87 | | 0.63, 1.19 | | | 0.378 | 0.97 | | 0.78, 1.21 | | | 0.799 | |  |
|  | Education level (low vs. high) | 0.90 | 0.74, 1.10 | | | 0.297 | 1.04 | | 0.84, 1.27 | | | 0.729 | 0.94 | | 0.69, 1.27 | | | 0.671 | 1.17 | | 0.91, 1.51 | | | 0.241 | |  |
|  | Household income (middle vs. high) | 1.25 | 0.98, 1.60 | | | 0.073 | 1.41 | | 1.08, 1.85 | | | 0.012 | 1.32 | | 0.89, 1.96 | | | 0.162 | 1.26 | | 0.95, 1.69 | | | 0.112 | |  |
|  | Household income (low vs. high) | 0.85 | 0.69, 1.05 | | | 0.131 | 1.36 | | 1.05, 1.77 | | | 0.020 | 1.18 | | 0.79, 1.74 | | | 0.421 | 1.11 | | 0.83, 1.47 | | | 0.487 | |  |
|  | Family history of MDD | 0.99 | 0.79, 1.25 | | | 0.944 | 1.37 | | 1.04, 1.82 | | | 0.026 | 1.24 | | 0.82, 1.88 | | | 0.314 | 1.51 | | 1.12, 2.03 | | | 0.007 | |  |
|  | Poor acceptance of pregnancy | 2.08 | 1.70, 2.53 | | | < 0.001 | 1.20 | | 0.93, 1.56 | | | 0.157 | 2.09 | | 1.55, 2.82 | | | < 0.001 | 2.52 | | 2.04, 3.13 | | | < 0.001 | |  |
|  | Social isolation | 1.21 | 1.03, 1.42 | | | 0.023 | 1.52 | | 1.27, 1.81 | | | < 0.001 | 1.37 | | 1.07, 1.76 | | | 0.014 | 2.01 | | 1.70, 2.38 | | | < 0.001 | |  |
|  | Unmarried | 1.43 | 0.93, 2.19 | | | 0.106 | 1.08 | | 0.62, 1.90 | | | 0.782 | 1.44 | | 0.72, 2.88 | | | 0.305 | 1.34 | | 0.82, 2.18 | | | 0.239 | |  |
|  | Neuroticism | 2.62 | 2.39, 2.88 | | | < 0.001 | 2.15 | | 1.97, 2.35 | | | < 0.001 | 2.04 | | 1.79, 2.33 | | | < 0.001 | 5.13 | | 4.60, 5.71 | | | < 0.001 | |  |
|  | Acquaintance deaths | 1.06 | 0.90, 1.25 | | | 0.511 | 1.00 | | 0.82, 1.22 | | | 0.993 | 1.32 | | 1.02, 1.71 | | | 0.038 | 1.10 | | 0.88, 1.38 | | | 0.406 | |  |
|  | House damages | 1.22 | 0.96, 1.55 | | | 0.116 | 1.04 | | 0.81, 1.34 | | | 0.755 | 1.05 | | 0.70, 1.57 | | | 0.821 | 0.95 | | 0.72, 1.26 | | | 0.746 | |  |
|  | Overweight | 0.91 | 0.74, 1.13 | | | 0.406 | 1.11 | | 0.90, 1.38 | | | 0.337 | 1.26 | | 0.93, 1.69 | | | 0.130 | 1.08 | | 0.86, 1.36 | | | 0.515 | |  |
|  | Male neonate | 1.00 | 0.88, 1.14 | | | 0.971 | 0.85 | | 0.73, 0.98 | | | 0.029 | 0.84 | | 0.68, 1.04 | | | 0.115 | 1.00 | | 0.85, 1.16 | | | 0.964 | |  |
|  | Primipara | 1.26 | 1.10, 1.44 | | | < 0.001 | 2.07 | | 1.78, 2.40 | | | < 0.001 | 0.90 | | 0.72, 1.12 | | | 0.349 | 1.36 | | 1.16, 1.59 | | | < 0.001 | |  |
|  | Multiple births | 1.14 | 0.62, 2.10 | | | 0.664 | 0.83 | | 0.39, 1.73 | | | 0.611 | 1.67 | | 0.72, 3.85 | | | 0.228 | 0.89 | | 0.40, 1.96 | | | 0.768 | |  |
|  | Caesarean section | 1.11 | 0.94, 1.30 | | | 0.213 | 1.09 | | 0.91, 1.30 | | | 0.348 | 0.92 | | 0.71, 1.20 | | | 0.536 | 1.07 | | 0.88, 1.30 | | | 0.483 | |  |
|  | Fetal growth restriction | 1.17 | 0.72, 1.91 | | | 0.521 | 1.24 | | 0.75, 2.06 | | | 0.399 | 1.73 | | 0.88, 3.40 | | | 0.111 | 0.81 | | 0.42, 1.55 | | | 0.518 | |  |
|  | Threatened prenatal delivery | 1.33 | 1.12, 1.58 | | | 0.001 | 1.37 | | 1.14, 1.66 | | | 0.001 | 1.14 | | 0.86, 1.51 | | | 0.355 | 1.15 | | 0.94, 1.41 | | | 0.187 | |  |
|  | Hypertensive disorders of pregnancy | 1.55 | 1.16, 2.07 | | | 0.003 | 1.29 | | 0.93, 1.79 | | | 0.133 | 1.25 | | 0.77, 2.03 | | | 0.365 | 1.06 | | 0.73, 1.54 | | | 0.766 | |  |
|  |  |  |  | | |  |  | |  | | |  |  | |  | | |  |  | |  | | |  | |  |
| Alternative outcome definition 1 | |  | |  |  | | |  | |  |  | | |  | |  |  | | |  | |  |  | |  |  |
|  | Age at delivery, years (30–35 vs. < 30) | 0.85 | 0.76, 0.95 | | | 0.005 | 1.47 | | 1.12, 1.93 | | | 0.006 | 0.86 | | 0.59, 1.26 | | | 0.442 | 0.82 | | 0.70, 0.96 | | | 0.016 | |  |
|  | Age at delivery, years (> 35 vs. < 30) | 0.78 | 0.69, 0.88 | | | < 0.001 | 1.10 | | 0.81, 1.49 | | | 0.530 | 0.87 | | 0.58, 1.31 | | | 0.510 | 0.68 | | 0.57, 0.82 | | | < 0.001 | |  |
|  | Education level (middle vs. high) | 0.95 | 0.83, 1.08 | | | 0.419 | 1.22 | | 0.91, 1.63 | | | 0.191 | 0.97 | | 0.62, 1.51 | | | 0.877 | 0.95 | | 0.79, 1.13 | | | 0.555 | |  |
|  | Education level (low vs. high) | 0.86 | 0.75, 0.98 | | | 0.022 | 1.11 | | 0.81, 1.52 | | | 0.503 | 0.94 | | 0.61, 1.44 | | | 0.768 | 1.01 | | 0.83, 1.23 | | | 0.936 | |  |
|  | Household income (middle vs. high) | 1.25 | 1.03, 1.53 | | | 0.031 | 1.37 | | 0.92, 2.06 | | | 0.122 | 1.67 | | 0.93, 3.01 | | | 0.089 | 1.50 | | 1.17, 1.94 | | | 0.002 | |  |
|  | Household income (low vs. high) | 1.11 | 0.96, 1.30 | | | 0.167 | 1.38 | | 0.96, 1.99 | | | 0.084 | 1.45 | | 0.83, 2.52 | | | 0.190 | 1.34 | | 1.05, 1.69 | | | 0.017 | |  |
|  | Family history of MDD | 1.19 | 0.99, 1.42 | | | 0.064 | 1.29 | | 0.86, 1.93 | | | 0.214 | 1.37 | | 0.76, 2.47 | | | 0.297 | 1.67 | | 1.28, 2.17 | | | < 0.001 | |  |
|  | Poor acceptance of pregnancy | 1.67 | 1.42, 1.96 | | | < 0.001 | 1.26 | | 0.85, 1.86 | | | 0.243 | 1.75 | | 1.08, 2.83 | | | 0.022 | 2.33 | | 1.88, 2.88 | | | < 0.001 | |  |
|  | Social isolation | 1.33 | 1.18, 1.50 | | | < 0.001 | 1.75 | | 1.35, 2.26 | | | < 0.001 | 1.49 | | 1.02, 2.16 | | | 0.038 | 1.93 | | 1.65, 2.26 | | | < 0.001 | |  |
|  | Unmarried | 1.34 | 0.94, 1.91 | | | 0.112 | 1.75 | | 0.87, 3.50 | | | 0.116 | 2.74 | | 1.24, 6.03 | | | 0.012 | 1.14 | | 0.70, 1.88 | | | 0.592 | |  |
|  | Neuroticism | 2.43 | 2.28, 2.60 | | | < 0.001 | 1.85 | | 1.63, 2.10 | | | < 0.001 | 1.75 | | 1.43, 2.14 | | | < 0.001 | 4.97 | | 4.52, 5.46 | | | < 0.001 | |  |
|  | Acquaintance deaths | 1.07 | 0.94, 1.21 | | | 0.294 | 0.92 | | 0.67, 1.27 | | | 0.613 | 1.39 | | 0.94, 2.04 | | | 0.097 | 1.08 | | 0.91, 1.29 | | | 0.392 | |  |
|  | House damages | 1.04 | 0.89, 1.21 | | | 0.629 | 1.05 | | 0.72, 1.55 | | | 0.793 | 1.10 | | 0.62, 1.94 | | | 0.741 | 0.97 | | 0.75, 1.23 | | | 0.778 | |  |
|  | Overweight | 1.04 | 0.90, 1.19 | | | 0.633 | 0.87 | | 0.62, 1.23 | | | 0.436 | 1.44 | | 0.95, 2.19 | | | 0.088 | 1.14 | | 0.92, 1.41 | | | 0.231 | |  |
|  | Male neonate | 0.96 | 0.88, 1.05 | | | 0.408 | 0.85 | | 0.68, 1.05 | | | 0.129 | 1.06 | | 0.78, 1.44 | | | 0.710 | 0.87 | | 0.76, 0.99 | | | 0.038 | |  |
|  | Primipara | 1.07 | 0.97, 1.18 | | | 0.177 | 2.03 | | 1.63, 2.53 | | | < 0.001 | 0.95 | | 0.68, 1.31 | | | 0.742 | 1.48 | | 1.29, 1.69 | | | < 0.001 | |  |
|  | Multiple births | 1.13 | 0.71, 1.79 | | | 0.605 | 0.91 | | 0.34, 2.43 | | | 0.848 | 2.09 | | 0.60, 7.25 | | | 0.247 | 0.87 | | 0.44, 1.73 | | | 0.695 | |  |
|  | Caesarean section | 1.02 | 0.91, 1.14 | | | 0.712 | 1.05 | | 0.81, 1.36 | | | 0.713 | 0.73 | | 0.49, 1.09 | | | 0.119 | 1.05 | | 0.89, 1.25 | | | 0.563 | |  |
|  | Fetal growth restriction | 1.20 | 0.84, 1.71 | | | 0.310 | 1.30 | | 0.61, 2.78 | | | 0.494 | 1.77 | | 0.63, 4.97 | | | 0.277 | 1.20 | | 0.72, 2.01 | | | 0.490 | |  |
|  | Threatened prenatal delivery | 1.28 | 1.13, 1.46 | | | < 0.001 | 1.55 | | 1.18, 2.04 | | | 0.002 | 1.09 | | 0.71, 1.67 | | | 0.700 | 1.31 | | 1.09, 1.58 | | | 0.004 | |  |
|  | Hypertensive disorders of pregnancy | 1.15 | 0.92, 1.45 | | | 0.212 | 1.80 | | 1.16, 2.77 | | | 0.008 | 1.36 | | 0.69, 2.68 | | | 0.377 | 1.03 | | 0.74, 1.43 | | | 0.856 | |  |
|  |  |  |  | | |  |  | |  | | |  |  | |  | | |  |  | |  | | |  | |  |
| Alternative outcome definition 2 | |  | |  |  | | |  | |  |  | | |  | |  |  | | |  | |  |  | | | |
|  | Age at delivery, years (30–35 vs. < 30) | 0.86 | 0.66, 1.12 | | | 0.267 | 1.13 | | 0.97, 1.31 | | | 0.120 | 0.87 | | 0.70, 1.08 | | | 0.208 | 0.71 | | 0.56, 0.91 | | | 0.007 | |  |
|  | Age at delivery, years (> 35 vs. < 30) | 0.80 | 0.60, 1.09 | | | 0.155 | 1.05 | | 0.88, 1.24 | | | 0.586 | 0.80 | | 0.63, 1.01 | | | 0.064 | 0.53 | | 0.39, 0.71 | | | < 0.001 | |  |
|  | Education level (middle vs. high) | 0.78 | 0.57, 1.08 | | | 0.137 | 1.04 | | 0.88, 1.22 | | | 0.681 | 0.84 | | 0.66, 1.09 | | | 0.193 | 1.14 | | 0.82, 1.59 | | | 0.442 | |  |
|  | Education level (low vs. high) | 0.97 | 0.71, 1.33 | | | 0.858 | 1.07 | | 0.90, 1.28 | | | 0.426 | 1.04 | | 0.81, 1.34 | | | 0.744 | 1.19 | | 0.83, 1.70 | | | 0.354 | |  |
|  | Household income (middle vs. high) | 1.46 | 0.95, 2.23 | | | 0.087 | 1.37 | | 1.08, 1.74 | | | 0.011 | 1.29 | | 0.92, 1.80 | | | 0.138 | 1.22 | | 0.82, 1.81 | | | 0.324 | |  |
|  | Household income (low vs. high) | 0.86 | 0.59, 1.24 | | | 0.413 | 1.49 | | 1.18, 1.89 | | | < 0.001 | 1.02 | | 0.75, 1.38 | | | 0.921 | 0.90 | | 0.60, 1.34 | | | 0.604 | |  |
|  | Family history of MDD | 1.12 | 0.76, 1.66 | | | 0.571 | 1.64 | | 1.28, 2.10 | | | < 0.001 | 1.06 | | 0.76, 1.48 | | | 0.725 | 1.29 | | 0.86, 1.95 | | | 0.224 | |  |
|  | Poor acceptance of pregnancy | 1.86 | 1.35, 2.55 | | | < 0.001 | 1.33 | | 1.09, 1.63 | | | 0.005 | 1.72 | | 1.33, 2.23 | | | < 0.001 | 2.83 | | 2.16, 3.71 | | | < 0.001 | |  |
|  | Social isolation | 1.17 | 0.90, 1.53 | | | 0.236 | 1.56 | | 1.35, 1.80 | | | < 0.001 | 1.52 | | 1.24, 1.86 | | | < 0.001 | 2.29 | | 1.83, 2.86 | | | < 0.001 | |  |
|  | Unmarried | 1.77 | 0.96, 3.26 | | | 0.069 | 1.09 | | 0.68, 1.73 | | | 0.724 | 1.40 | | 0.78, 2.52 | | | 0.258 | 1.63 | | 0.92, 2.87 | | | 0.094 | |  |
|  | Neuroticism | 2.63 | 2.27, 3.06 | | | < 0.001 | 2.39 | | 2.21, 2.59 | | | < 0.001 | 2.20 | | 1.96, 2.46 | | | < 0.001 | 5.49 | | 4.70, 6.41 | | | < 0.001 | |  |
|  | Acquaintance deaths | 1.02 | 0.74, 1.40 | | | 0.898 | 0.97 | | 0.81, 1.17 | | | 0.779 | 1.32 | | 1.06, 1.63 | | | 0.012 | 1.19 | | 0.89, 1.59 | | | 0.244 | |  |
|  | House damages | 1.49 | 1.07, 2.08 | | | 0.019 | 1.02 | | 0.82, 1.26 | | | 0.888 | 0.93 | | 0.66, 1.31 | | | 0.685 | 0.97 | | 0.67, 1.42 | | | 0.882 | |  |
|  | Overweight | 0.80 | 0.55, 1.16 | | | 0.236 | 1.12 | | 0.94, 1.35 | | | 0.212 | 1.09 | | 0.84, 1.42 | | | 0.501 | 1.20 | | 0.89, 1.62 | | | 0.235 | |  |
|  | Male neonate | 1.06 | 0.85, 1.33 | | | 0.582 | 0.85 | | 0.75, 0.97 | | | 0.013 | 0.88 | | 0.74, 1.05 | | | 0.164 | 1.08 | | 0.88, 1.34 | | | 0.461 | |  |
|  | Primipara | 1.17 | 0.93, 1.47 | | | 0.189 | 1.87 | | 1.64, 2.12 | | | < 0.001 | 0.87 | | 0.72, 1.06 | | | 0.163 | 1.18 | | 0.95, 1.46 | | | 0.141 | |  |
|  | Multiple births | 0.19 | 0.03, 1.42 | | | 0.106 | 0.75 | | 0.39, 1.42 | | | 0.375 | 1.27 | | 0.58, 2.74 | | | 0.551 | 1.39 | | 0.53, 3.64 | | | 0.502 | |  |
|  | Caesarean section | 1.18 | 0.90, 1.53 | | | 0.227 | 1.08 | | 0.93, 1.25 | | | 0.330 | 0.96 | | 0.77, 1.19 | | | 0.682 | 0.97 | | 0.74, 1.27 | | | 0.835 | |  |
|  | Fetal growth restriction | 1.27 | 0.60, 2.71 | | | 0.536 | 1.15 | | 0.73, 1.79 | | | 0.548 | 1.46 | | 0.80, 2.65 | | | 0.214 | 0.50 | | 0.18, 1.37 | | | 0.176 | |  |
|  | Threatened prenatal delivery | 1.45 | 1.10, 1.92 | | | 0.008 | 1.26 | | 1.07, 1.48 | | | 0.006 | 1.15 | | 0.91, 1.46 | | | 0.229 | 1.08 | | 0.81, 1.44 | | | 0.606 | |  |
|  | Hypertensive disorders of pregnancy | 1.61 | 1.02, 2.54 | | | 0.042 | 1.17 | | 0.88, 1.57 | | | 0.285 | 1.10 | | 0.72, 1.69 | | | 0.649 | 1.25 | | 0.76, 2.06 | | | 0.370 | |  |
|  |  |  |  | | |  |  | |  | | |  |  | |  | | |  |  | |  | | |  | |  |
| Alternative population | |  | |  |  | | |  | |  |  | | |  | |  |  | | |  | |  |  | | | |
|  | Age at delivery, years (30–35 vs. < 30) | 0.82 | 0.70, 0.96 | | | 0.015 | 1.16 | | 0.97, 1.39 | | | 0.108 | 0.84 | | 0.65, 1.09 | | | 0.199 | 0.84 | | 0.70, 1.01 | | | 0.058 | |  |
|  | Age at delivery, years (> 35 vs. < 30) | 0.72 | 0.60, 0.86 | | | < 0.001 | 1.03 | | 0.84, 1.26 | | | 0.805 | 0.80 | | 0.60, 1.06 | | | 0.120 | 0.72 | | 0.58, 0.89 | | | 0.002 | |  |
|  | Education level (middle vs. high) | 0.95 | 0.79, 1.14 | | | 0.596 | 1.06 | | 0.87, 1.30 | | | 0.543 | 0.86 | | 0.63, 1.18 | | | 0.368 | 0.98 | | 0.79, 1.23 | | | 0.881 | |  |
|  | Education level (low vs. high) | 0.89 | 0.73, 1.09 | | | 0.254 | 1.03 | | 0.84, 1.27 | | | 0.755 | 0.95 | | 0.70, 1.28 | | | 0.718 | 1.17 | | 0.91, 1.52 | | | 0.234 | |  |
|  | Household income (middle vs. high) | 1.26 | 0.99, 1.61 | | | 0.065 | 1.43 | | 1.09, 1.87 | | | 0.009 | 1.35 | | 0.91, 1.99 | | | 0.138 | 1.28 | | 0.96, 1.71 | | | 0.091 | |  |
|  | Household income (low vs. high) | 0.84 | 0.67, 1.03 | | | 0.099 | 1.39 | | 1.07, 1.81 | | | 0.014 | 1.19 | | 0.80, 1.78 | | | 0.389 | 1.09 | | 0.82, 1.45 | | | 0.543 | |  |
|  | Family history of MDD | 0.99 | 0.79, 1.25 | | | 0.945 | 1.41 | | 1.06, 1.86 | | | 0.018 | 1.25 | | 0.82, 1.91 | | | 0.299 | 1.49 | | 1.10, 2.01 | | | 0.010 | |  |
|  | Poor acceptance of pregnancy | 2.05 | 1.68, 2.50 | | | < 0.001 | 1.19 | | 0.92, 1.54 | | | 0.183 | 2.11 | | 1.56, 2.85 | | | < 0.001 | 2.50 | | 2.02, 3.10 | | | < 0.001 | |  |
|  | Social isolation | 1.20 | 1.02, 1.42 | | | 0.026 | 1.51 | | 1.27, 1.80 | | | < 0.001 | 1.35 | | 1.05, 1.73 | | | 0.021 | 2.00 | | 1.69, 2.37 | | | < 0.001 | |  |
|  | Unmarried | 1.45 | 0.95, 2.22 | | | 0.087 | 1.09 | | 0.62, 1.90 | | | 0.771 | 1.46 | | 0.73, 2.92 | | | 0.290 | 1.35 | | 0.83, 2.20 | | | 0.228 | |  |
|  | Neuroticism | 2.62 | 2.39, 2.88 | | | < 0.001 | 2.15 | | 1.97, 2.35 | | | < 0.001 | 2.06 | | 1.79, 2.36 | | | < 0.001 | 5.10 | | 4.58, 5.68 | | | < 0.001 | |  |
|  | Acquaintance deaths | 1.06 | 0.90, 1.25 | | | 0.502 | 1.00 | | 0.82, 1.22 | | | 0.997 | 1.32 | | 1.02, 1.72 | | | 0.038 | 1.10 | | 0.88, 1.38 | | | 0.421 | |  |
|  | House damages | 1.20 | 0.94, 1.52 | | | 0.148 | 1.05 | | 0.82, 1.35 | | | 0.713 | 1.03 | | 0.69, 1.55 | | | 0.872 | 0.95 | | 0.72, 1.26 | | | 0.748 | |  |
|  | Overweight | 0.92 | 0.75, 1.14 | | | 0.459 | 1.13 | | 0.91, 1.40 | | | 0.269 | 1.29 | | 0.96, 1.74 | | | 0.093 | 1.10 | | 0.87, 1.38 | | | 0.417 | |  |
|  | Male neonate | 1.02 | 0.89, 1.16 | | | 0.818 | 0.85 | | 0.74, 0.99 | | | 0.036 | 0.85 | | 0.69, 1.05 | | | 0.138 | 1.00 | | 0.86, 1.17 | | | 0.951 | |  |
|  | Primipara | 1.25 | 1.09, 1.44 | | | 0.001 | 2.06 | | 1.77, 2.40 | | | < 0.001 | 0.89 | | 0.71, 1.12 | | | 0.322 | 1.36 | | 1.16, 1.59 | | | < 0.001 | |  |
|  | Multiple births | 1.15 | 0.63, 2.11 | | | 0.656 | 0.83 | | 0.40, 1.74 | | | 0.624 | 1.70 | | 0.73, 3.92 | | | 0.216 | 0.90 | | 0.41, 1.98 | | | 0.785 | |  |
|  | Caesarean section | 1.13 | 0.96, 1.32 | | | 0.139 | 1.09 | | 0.91, 1.30 | | | 0.352 | 0.92 | | 0.71, 1.20 | | | 0.536 | 1.08 | | 0.89, 1.30 | | | 0.454 | |  |
|  | Fetal growth restriction | 1.22 | 0.75, 1.99 | | | 0.418 | 1.28 | | 0.77, 2.13 | | | 0.336 | 1.60 | | 0.79, 3.25 | | | 0.193 | 0.83 | | 0.43, 1.60 | | | 0.581 | |  |
|  | Threatened prenatal delivery | 1.34 | 1.13, 1.59 | | | < 0.001 | 1.37 | | 1.13, 1.65 | | | 0.001 | 1.13 | | 0.85, 1.51 | | | 0.381 | 1.13 | | 0.92, 1.40 | | | 0.231 | |  |
|  | Hypertensive disorders of pregnancy | 1.48 | 1.10, 1.99 | | | 0.010 | 1.29 | | 0.93, 1.79 | | | 0.130 | 1.21 | | 0.74, 1.98 | | | 0.456 | 1.05 | | 0.72, 1.53 | | | 0.787 | |  |

PD: Perinatal depression, OR: Odds ratio, CI: Confidence interval, MDD: Major depressive disorder.

Multinomial logistic regression model included all variables in this table.

The cutoff for K6 was 9 in the main definition, 5 in alternative outcome definition 1, and 13 in alternative outcome definition 2.

In the alternative population, those who got pregnant at the time they responded to the K6 at 6 months postpartum were excluded from the study population.

Supplementary Table 3.Association of six GWAS significant or top hitvariants with PD subtypes

|  |  | JPA v2 | | | |  | JPA NEO | | | |  | Meta-analysis | |
| --- | --- | --- | --- | --- | --- | --- | --- | --- | --- | --- | --- | --- | --- |
|  | Subtypes | EA freq. | Beta | SE | P-value |  | EA freq. | Beta | SE | P-value |  | P-value | Direction |
| rs138801403 | |  |  |  |  |  |  |  |  |  |  |  |  |
|  | **Any PD** | **0.012** | **0.840** | **0.190** | **5.8 × 10^-6^** |  | **0.013** | **0.640** | **0.200** | **1.4 × 10^-3^** |  | **3.4 × 10^-8^** | **++** |
|  | Pregnancy PD | 0.010 | 1.370 | 0.330 | 3.6 × 10^-5^ |  | 0.012 | 0.810 | 0.330 | 1.4 × 10^-2^ |  | 2.3 × 10^-6^ | ++ |
|  | Early postpartum PD | 0.010 | 0.730 | 0.350 | 3.6 × 10^-2^ |  | 0.011 | 0.710 | 0.390 | 6.7 × 10^-2^ |  | 5.4 × 10^-3^ | ++ |
|  | Late postpartum PD | 0.009 | 0.370 | 0.530 | 4.8 × 10^-1^ |  | 0.011 | 0.810 | 0.560 | 1.5 × 10^-1^ |  | 1.4 × 10^-1^ | ++ |
|  | Chronic PD | 0.010 | 0.990 | 0.330 | 2.9 × 10^-3^ |  | 0.011 | 0.670 | 0.340 | 4.7 × 10^-2^ |  | 3.9 × 10^-4^ | ++ |
| rs1853229 | |  |  |  |  |  |  |  |  |  |  |  |  |
|  | Any PD | 0.021 | 0.420 | 0.140 | 1.9 × 10^-3^ |  | 0.022 | 0.540 | 0.160 | 4.7 × 10^-4^ |  | 3.5 × 10^-6^ | ++ |
|  | **Pregnancy PD** | **0.021** | **0.900** | **0.220** | **4.5 × 10^-5^** |  | **0.021** | **0.980** | **0.260** | **1.2 × 10^-4^** |  | **2.1 × 10^-8^** | **++** |
|  | Early postpartum PD | 0.019 | -0.040 | 0.250 | 8.8 × 10^-1^ |  | 0.019 | 0.100 | 0.310 | 7.5 × 10^-1^ |  | 9.2 × 10^-1^ | -+ |
|  | Late postpartum PD | 0.019 | 0.070 | 0.370 | 8.5 × 10^-1^ |  | 0.020 | 1.440 | 0.460 | 1.6 × 10^-3^ |  | 2.6 × 10^-2^ | ++ |
|  | Chronic PD | 0.020 | 0.440 | 0.230 | 5.8 × 10^-2^ |  | 0.019 | 0.260 | 0.270 | 3.3 × 10^-1^ |  | 3.9 × 10^-2^ | ++ |
| rs117741236 | |  |  |  |  |  |  |  |  |  |  |  |  |
|  | Any PD | 0.022 | 0.270 | 0.130 | 4.0 × 10^-2^ |  | 0.027 | 0.230 | 0.140 | 9.9 × 10^-2^ |  | 8.6 × 10^-3^ | ++ |
|  | Pregnancy PD | 0.021 | -0.020 | 0.220 | 9.1 × 10^-1^ |  | 0.025 | -0.160 | 0.230 | 4.9 × 10^-1^ |  | 5.9 × 10^-1^ | -- |
|  | **Early postpartum PD** | **0.023** | **1.010** | **0.240** | **3.2 × 10^-5^** |  | **0.027** | **0.900** | **0.260** | **5.5 × 10^-4^** |  | **6.5 × 10^-8^** | **++** |
|  | Late postpartum PD | 0.020 | -0.440 | 0.360 | 2.2 × 10^-1^ |  | 0.025 | 0.000 | 0.370 | 9.9 × 10^-1^ |  | 3.5 × 10^-1^ | -- |
|  | Chronic PD | 0.021 | 0.170 | 0.220 | 4.4 × 10^-1^ |  | 0.026 | 0.160 | 0.230 | 4.8 × 10^-1^ |  | 2.9 × 10^-1^ | ++ |
| rs1075046 | |  |  |  |  |  |  |  |  |  |  |  |  |
|  | Any PD | 0.549 | -0.010 | 0.040 | 7.1 × 10^-1^ |  | 0.552 | -0.060 | 0.040 | 2.1 × 10^-1^ |  | 2.7 × 10^-1^ | -- |
|  | Pregnancy PD | 0.551 | 0.040 | 0.060 | 5.2 × 10^-1^ |  | 0.557 | 0.060 | 0.070 | 3.8 × 10^-1^ |  | 2.9 × 10^-1^ | ++ |
|  | Early postpartum PD | 0.551 | 0.080 | 0.070 | 2.6 × 10^-1^ |  | 0.554 | -0.060 | 0.080 | 4.8 × 10^-1^ |  | 7.1 × 10^-1^ | +- |
|  | **Late postpartum PD** | **0.546** | **-0.310** | **0.100** | **2.2 × 10^-3^** |  | **0.549** | **-0.630** | **0.120** | **5.2 × 10^-8^** |  | **3.7 × 10^-9^** | **--** |
|  | Chronic PD | 0.549 | -0.050 | 0.070 | 4.8 × 10^-1^ |  | 0.556 | 0.010 | 0.070 | 8.4 × 10^-1^ |  | 6.9 × 10^-1^ | -+ |
| rs4786119 | |  |  |  |  |  |  |  |  |  |  |  |  |
|  | Any PD | 0.615 | -0.070 | 0.040 | 9.4 × 10^-2^ |  | 0.611 | -0.070 | 0.050 | 1.5 × 10^-1^ |  | 2.8 × 10^-2^ | -- |
|  | Pregnancy PD | 0.620 | 0.040 | 0.060 | 4.9 × 10^-1^ |  | 0.616 | 0.030 | 0.070 | 7.1 × 10^-1^ |  | 4.4 × 10^-1^ | ++ |
|  | Early postpartum PD | 0.617 | -0.100 | 0.070 | 1.7 × 10^-1^ |  | 0.613 | -0.090 | 0.090 | 2.8 × 10^-1^ |  | 8.1 × 10^-2^ | -- |
|  | **Late postpartum PD** | **0.615** | **-0.380** | **0.110** | **3.2 × 10^-4^** |  | **0.609** | **-0.570** | **0.120** | **2.3 × 10^-6^** |  | **5.8 × 10^-9^** | **--** |
|  | Chronic PD | 0.618 | -0.050 | 0.070 | 4.5 × 10^-1^ |  | 0.616 | 0.040 | 0.080 | 5.7 × 10^-1^ |  | 8.4 × 10^-1^ | -+ |
| rs56289435 | |  |  |  |  |  |  |  |  |  |  |  |  |
|  | Any PD | 0.500 | -0.050 | 0.040 | 2.4 × 10^-1^ |  | 0.500 | -0.150 | 0.050 | 1.2 × 10^-3^ |  | 2.5 × 10^-3^ | -- |
|  | Pregnancy PD | 0.503 | -0.020 | 0.060 | 7.4 × 10^-1^ |  | 0.509 | -0.050 | 0.070 | 4.6 × 10^-1^ |  | 4.6 × 10^-1^ | -- |
|  | Early postpartum PD | 0.506 | 0.090 | 0.070 | 1.9 × 10^-1^ |  | 0.509 | -0.030 | 0.080 | 7.1 × 10^-1^ |  | 4.7 × 10^-1^ | +- |
|  | Late postpartum PD | 0.503 | -0.060 | 0.110 | 5.9 × 10^-1^ |  | 0.509 | -0.070 | 0.120 | 5.3 × 10^-1^ |  | 4.1 × 10^-1^ | -- |
|  | **Chronic PD** | **0.498** | **-0.200** | **0.070** | **3.1 × 10^-3^** |  | **0.500** | **-0.370** | **0.070** | **7.2 × 10^-7^** |  | **3.9 × 10^-8^** | **--** |

PD: Perinatal depression, JPA v2: Japonica Array v2, JPA NEO: Japonica Array NEO.

EA freq: Effect allele frequency, SE: Standard error.

Supplementary Table 4. Genetic correlations between PD subtypes

|  | Any PD | Pregnancy PD | Early postpartum PD | Late postpartum PD |
| --- | --- | --- | --- | --- |
| Pregnancy PD | NA |  |  |  |
| Early postpartum PD | NA | NA |  |  |
| Late postpartum PD | 0.34 (0.38), P = 0.370 | 0.62 (0.78), P = 0.429 | NA |  |
| Chronic PD | 0.73 (0.36), P = 0.042 | 0.94 (0.98), P = 0.338 | NA | -0.33 (0.62), P = 0.590 |

PD: Perinatal depression.

Each cell representsgenetic correlations, standard error, and P-value.

Cells with NA were not calculated because the heritability is small, or the calculation did not converge.

Supplementary Table 4. Relationship between polygenic risk score for major depressive disorder and premenstrual syndrome, and PD subtypes

|  |  | Major depressive disorder^a^ | | |  | Premenstrual syndrome^b^ | | |  | Major depressive disorder^c^ | | |
| --- | --- | --- | --- | --- | --- | --- | --- | --- | --- | --- | --- | --- |
|  |  | OR | 95% CI | P for trend |  | OR | 95% CI | P for trend |  | OR | 95% CI | P for trend |
|  |  |  |  |  |  |  |  |  |  |  |  |  |
| Main definition | |  |  |  |  |  |  |  |  |  |  |  |
|  | Pregnancy PD | 1.00 | Reference | 0.018 |  | 1.00 | Reference | 0.600 |  | 1.00 | Reference | 0.333 |
|  |  | 1.00 | 0.82, 1.23 |  |  | 0.96 | 0.78, 1.18 |  |  | 0.97 | 0.79, 1.19 |  |
|  |  | 1.17 | 0.96, 1.43 |  |  | 0.94 | 0.77, 1.15 |  |  | 1.00 | 0.82, 1.23 |  |
|  |  | 1.32 | 1.08, 1.60 |  |  | 0.89 | 0.73, 1.10 |  |  | 1.10 | 0.91, 1.35 |  |
|  |  | 1.14 | 0.93, 1.40 |  |  | 1.09 | 0.89, 1.34 |  |  | 1.05 | 0.86, 1.28 |  |
|  | Early postpartum PD | 1.00 | Reference | 0.012 |  | 1.00 | Reference | 0.998 |  | 1.00 | Reference | 0.082 |
|  |  | 1.03 | 0.81, 1.30 |  |  | 0.97 | 0.77, 1.23 |  |  | 0.95 | 0.75, 1.20 |  |
|  |  | 1.22 | 0.97, 1.53 |  |  | 1.07 | 0.85, 1.35 |  |  | 1.13 | 0.90, 1.42 |  |
|  |  | 1.15 | 0.91, 1.45 |  |  | 0.94 | 0.74, 1.19 |  |  | 0.98 | 0.78, 1.24 |  |
|  |  | 1.30 | 1.04, 1.63 |  |  | 1.02 | 0.81, 1.29 |  |  | 1.22 | 0.98, 1.53 |  |
|  | Late postpartum PD | 1.00 | Reference | 0.103 |  | 1.00 | Reference | 0.832 |  | 1.00 | Reference | 0.537 |
|  |  | 0.83 | 0.59, 1.18 |  |  | 0.96 | 0.67, 1.35 |  |  | 1.03 | 0.74, 1.43 |  |
|  |  | 0.98 | 0.70, 1.37 |  |  | 1.09 | 0.78, 1.52 |  |  | 1.27 | 0.92, 1.75 |  |
|  |  | 1.12 | 0.81, 1.55 |  |  | 1.06 | 0.76, 1.49 |  |  | 1.07 | 0.77, 1.49 |  |
|  |  | 1.18 | 0.86, 1.63 |  |  | 0.99 | 0.70, 1.40 |  |  | 0.85 | 0.60, 1.21 |  |
|  | Chronic PD | 1.00 | Reference | < 0.001 |  | 1.00 | Reference | 0.908 |  | 1.00 | Reference | 0.177 |
|  |  | 1.13 | 0.90, 1.41 |  |  | 0.95 | 0.76, 1.17 |  |  | 0.96 | 0.78, 1.19 |  |
|  |  | 1.44 | 1.16, 1.78 |  |  | 0.99 | 0.80, 1.22 |  |  | 1.08 | 0.88, 1.33 |  |
|  |  | 1.41 | 1.13, 1.74 |  |  | 1.05 | 0.85, 1.29 |  |  | 1.14 | 0.92, 1.40 |  |
|  |  | 1.50 | 1.21, 1.85 |  |  | 0.95 | 0.77, 1.18 |  |  | 1.08 | 0.88, 1.33 |  |
|  |  |  |  |  |  |  |  |  |  |  |  |  |
| Alternative outcome definition 1 | |  |  |  |  |  |  |  |  |  |  |  |
|  | Pregnancy PD | 1.00 | Reference | 0.068 |  | 1.00 | Reference | 0.133 |  | 1.00 | Reference | 0.551 |
|  |  | 1.06 | 0.92, 1.21 |  |  | 1.09 | 0.95, 1.25 |  |  | 1.00 | 0.88, 1.15 |  |
|  |  | 1.05 | 0.92, 1.20 |  |  | 1.08 | 0.94, 1.24 |  |  | 1.09 | 0.95, 1.25 |  |
|  |  | 1.09 | 0.95, 1.25 |  |  | 1.05 | 0.91, 1.20 |  |  | 1.03 | 0.90, 1.18 |  |
|  |  | 1.13 | 0.99, 1.29 |  |  | 1.15 | 1.00, 1.32 |  |  | 1.03 | 0.90, 1.18 |  |
|  | Early postpartum PD | 1.00 | Reference | 0.193 |  | 1.00 | Reference | 0.191 |  | 1.00 | Reference | 0.311 |
|  |  | 0.95 | 0.67, 1.35 |  |  | 0.96 | 0.67, 1.36 |  |  | 1.04 | 0.73, 1.48 |  |
|  |  | 1.19 | 0.85, 1.66 |  |  | 1.07 | 0.76, 1.52 |  |  | 1.36 | 0.98, 1.91 |  |
|  |  | 1.20 | 0.86, 1.68 |  |  | 1.12 | 0.79, 1.58 |  |  | 1.08 | 0.76, 1.54 |  |
|  |  | 1.15 | 0.82, 1.61 |  |  | 1.19 | 0.84, 1.68 |  |  | 1.19 | 0.84, 1.68 |  |
|  | Late postpartum PD | 1.00 | Reference | 0.190 |  | 1.00 | Reference | 0.917 |  | 1.00 | Reference | 0.738 |
|  |  | 1.05 | 0.64, 1.72 |  |  | 1.24 | 0.75, 2.07 |  |  | 1.42 | 0.87, 2.31 |  |
|  |  | 0.91 | 0.55, 1.53 |  |  | 1.23 | 0.74, 2.03 |  |  | 1.35 | 0.81, 2.24 |  |
|  |  | 1.27 | 0.78, 2.05 |  |  | 1.17 | 0.71, 1.94 |  |  | 1.33 | 0.81, 2.17 |  |
|  |  | 1.30 | 0.81, 2.10 |  |  | 1.02 | 0.59, 1.74 |  |  | 0.93 | 0.54, 1.61 |  |
|  | Chronic PD | 1.00 | Reference | < 0.001 |  | 1.00 | Reference | 0.934 |  | 1.00 | Reference | 0.119 |
|  |  | 1.07 | 0.90, 1.29 |  |  | 0.98 | 0.82, 1.16 |  |  | 0.92 | 0.77, 1.10 |  |
|  |  | 1.31 | 1.10, 1.56 |  |  | 1.05 | 0.89, 1.25 |  |  | 1.11 | 0.93, 1.32 |  |
|  |  | 1.26 | 1.05, 1.50 |  |  | 1.01 | 0.85, 1.20 |  |  | 1.04 | 0.88, 1.23 |  |
|  |  | 1.47 | 1.24, 1.75 |  |  | 0.99 | 0.83, 1.18 |  |  | 1.10 | 0.93, 1.31 |  |
|  |  |  |  |  |  |  |  |  |  |  |  |  |
| Alternative outcome definition 2 | |  |  |  |  |  |  |  |  |  |  |  |
|  | Pregnancy PD | 1.00 | Reference | 0.491 |  | 1.00 | Reference | 0.600 |  | 1.00 | Reference | 0.992 |
|  |  | 0.94 | 0.66, 1.34 |  |  | 1.20 | 0.85, 1.69 |  |  | 0.91 | 0.64, 1.29 |  |
|  |  | 1.05 | 0.74, 1.49 |  |  | 0.90 | 0.62, 1.30 |  |  | 0.92 | 0.65, 1.31 |  |
|  |  | 1.17 | 0.84, 1.65 |  |  | 0.91 | 0.63, 1.31 |  |  | 0.99 | 0.70, 1.40 |  |
|  |  | 1.03 | 0.72, 1.46 |  |  | 1.05 | 0.73, 1.50 |  |  | 0.96 | 0.68, 1.36 |  |
|  | Early postpartum PD | 1.00 | Reference | < 0.001 |  | 1.00 | Reference | 0.873 |  | 1.00 | Reference | 0.047 |
|  |  | 1.04 | 0.85, 1.27 |  |  | 1.02 | 0.84, 1.23 |  |  | 0.96 | 0.79, 1.17 |  |
|  |  | 1.26 | 1.04, 1.52 |  |  | 1.04 | 0.85, 1.26 |  |  | 1.15 | 0.95, 1.39 |  |
|  |  | 1.26 | 1.04, 1.53 |  |  | 1.02 | 0.84, 1.24 |  |  | 1.02 | 0.84, 1.24 |  |
|  |  | 1.31 | 1.08, 1.59 |  |  | 0.98 | 0.81, 1.19 |  |  | 1.20 | 0.99, 1.44 |  |
|  | Late postpartum PD | 1.00 | Reference | 0.142 |  | 1.00 | Reference | 0.694 |  | 1.00 | Reference | 0.951 |
|  |  | 0.95 | 0.71, 1.26 |  |  | 0.90 | 0.67, 1.21 |  |  | 1.03 | 0.78, 1.36 |  |
|  |  | 1.00 | 0.75, 1.33 |  |  | 1.08 | 0.81, 1.42 |  |  | 1.21 | 0.92, 1.59 |  |
|  |  | 1.16 | 0.89, 1.53 |  |  | 0.98 | 0.74, 1.31 |  |  | 1.02 | 0.77, 1.36 |  |
|  |  | 1.14 | 0.87, 1.50 |  |  | 1.02 | 0.77, 1.36 |  |  | 0.99 | 0.74, 1.32 |  |
|  | Chronic PD | 1.00 | Reference | < 0.001 |  | 1.00 | Reference | 0.934 |  | 1.00 | Reference | 0.812 |
|  |  | 1.13 | 0.81, 1.57 |  |  | 0.93 | 0.69, 1.27 |  |  | 0.93 | 0.68, 1.26 |  |
|  |  | 1.55 | 1.14, 2.11 |  |  | 1.01 | 0.75, 1.38 |  |  | 0.98 | 0.72, 1.34 |  |
|  |  | 1.18 | 0.85, 1.64 |  |  | 1.07 | 0.79, 1.45 |  |  | 1.16 | 0.87, 1.56 |  |
|  |  | 1.74 | 1.28, 2.35 |  |  | 0.91 | 0.66, 1.25 |  |  | 0.92 | 0.68, 1.25 |  |
|  |  |  |  |  |  |  |  |  |  |  |  |  |
| Alternative population | |  |  |  |  |  |  |  |  |  |  |  |
|  | Pregnancy PD | 1.00 | Reference | 0.649 |  | 1.00 | Reference | 0.667 |  | 1.00 | Reference | 0.966 |
|  |  | 0.93 | 0.65, 1.32 |  |  | 1.22 | 0.86, 1.73 |  |  | 0.92 | 0.64, 1.30 |  |
|  |  | 1.04 | 0.73, 1.48 |  |  | 0.89 | 0.62, 1.30 |  |  | 0.93 | 0.66, 1.33 |  |
|  |  | 1.09 | 0.77, 1.55 |  |  | 0.93 | 0.64, 1.34 |  |  | 1.02 | 0.72, 1.44 |  |
|  |  | 1.01 | 0.71, 1.44 |  |  | 1.07 | 0.74, 1.53 |  |  | 0.94 | 0.66, 1.34 |  |
|  | Early postpartum PD | 1.00 | Reference | < 0.001 |  | 1.00 | Reference | 0.899 |  | 1.00 | Reference | 0.047 |
|  |  | 1.04 | 0.85, 1.27 |  |  | 1.02 | 0.84, 1.24 |  |  | 0.95 | 0.78, 1.16 |  |
|  |  | 1.25 | 1.03, 1.52 |  |  | 1.04 | 0.86, 1.26 |  |  | 1.14 | 0.95, 1.38 |  |
|  |  | 1.27 | 1.05, 1.54 |  |  | 1.02 | 0.84, 1.25 |  |  | 1.02 | 0.84, 1.23 |  |
|  |  | 1.33 | 1.10, 1.61 |  |  | 0.98 | 0.81, 1.20 |  |  | 1.20 | 0.99, 1.44 |  |
|  | Late postpartum PD | 1.00 | Reference | 0.135 |  | 1.00 | Reference | 0.764 |  | 1.00 | Reference | 0.971 |
|  |  | 0.93 | 0.70, 1.23 |  |  | 0.89 | 0.67, 1.20 |  |  | 1.06 | 0.80, 1.40 |  |
|  |  | 0.97 | 0.73, 1.29 |  |  | 1.06 | 0.80, 1.40 |  |  | 1.24 | 0.94, 1.63 |  |
|  |  | 1.15 | 0.88, 1.51 |  |  | 0.98 | 0.74, 1.30 |  |  | 1.04 | 0.78, 1.39 |  |
|  |  | 1.14 | 0.87, 1.50 |  |  | 1.00 | 0.76, 1.34 |  |  | 1.01 | 0.76, 1.35 |  |
|  | Chronic PD | 1.00 | Reference | < 0.001 |  | 1.00 | Reference | 0.957 |  | 1.00 | Reference | 0.653 |
|  |  | 1.14 | 0.82, 1.60 |  |  | 0.92 | 0.68, 1.26 |  |  | 0.91 | 0.67, 1.24 |  |
|  |  | 1.59 | 1.16, 2.18 |  |  | 1.01 | 0.74, 1.37 |  |  | 0.99 | 0.73, 1.36 |  |
|  |  | 1.23 | 0.88, 1.71 |  |  | 1.07 | 0.79, 1.45 |  |  | 1.18 | 0.88, 1.58 |  |
|  |  | 1.79 | 1.32, 2.44 |  |  | 0.91 | 0.66, 1.25 |  |  | 0.94 | 0.69, 1.28 |  |

PD: Perinatal depression, OR: Odds ratio, CI: Confidence interval.

The cutoff for K6 was 9 in the main definition, 5 in alternative definition 1, and 13 in alternative definition 2.

In the alternative population, those who got pregnant at the time they responded to the K6 at 6 months postpartum were excluded from the study population.

All models included age and first five genetic principal components as covariates.

^a^Derived from the European population in the Psychiatric Genomics Consortium.

^b^Derived from the Japanese population in the Tohoku Medical Megabank Organization.

^c^Derived from the meta-analysis including the Japanese population in the BioBank Japan.
